# Supplementary material for: Multistaged discharge constructing heterostructure with enhanced solid-solution behavior for long-life lithium-oxygen batteries
Source: Nat Commun. 2019 Dec 20;10:5810. doi: 10.1038/s41467-019-13712-2 (PMC6925149; doi:10.1038/s41467-019-13712-2)
Supplement: Supplementary file 1 — Supplementary Information [file 41467_2019_13712_MOESM1_ESM.pdf]

Supplementary information

Multistaged discharge constructing heterostructure with enhanced solid-solution behavior for long-life lithium-oxygen batteries

Xu et al.

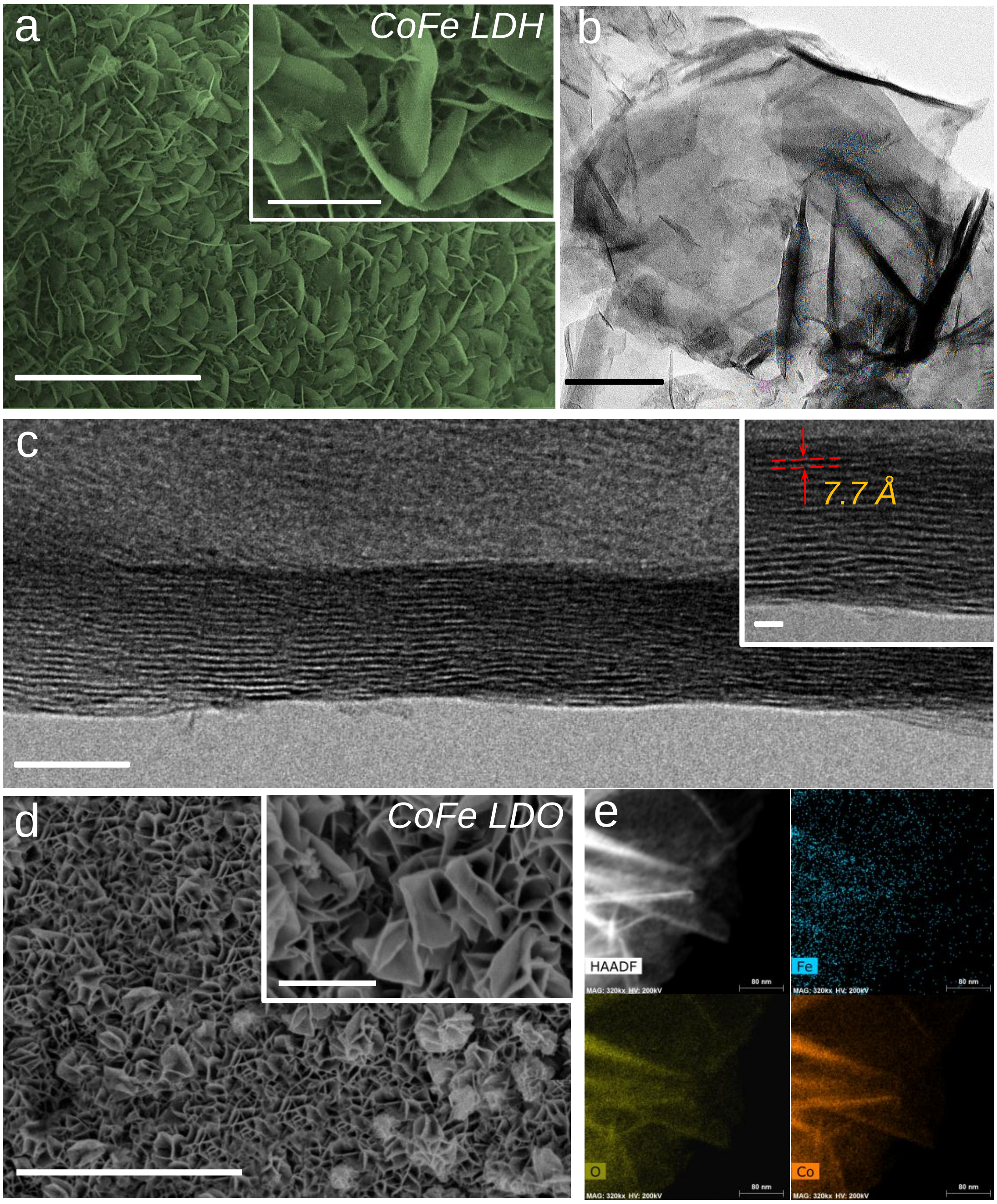

Supplementary Figure 1 | Characterization of CoFe LDH and CoFe LDO. **a-c**, SEM and TEM images of CoFe LDH arrays. **d**,**e**, SEM and elemental mapping images of CoFe LDO. Scale bars of **a-d** are 5 µm, 200 nm, 10 nm and 5 µm, respectively. Scale bars of insets in **a**,**c**,**d** are 1 µm, 2 nm and 1 µm, respectively.

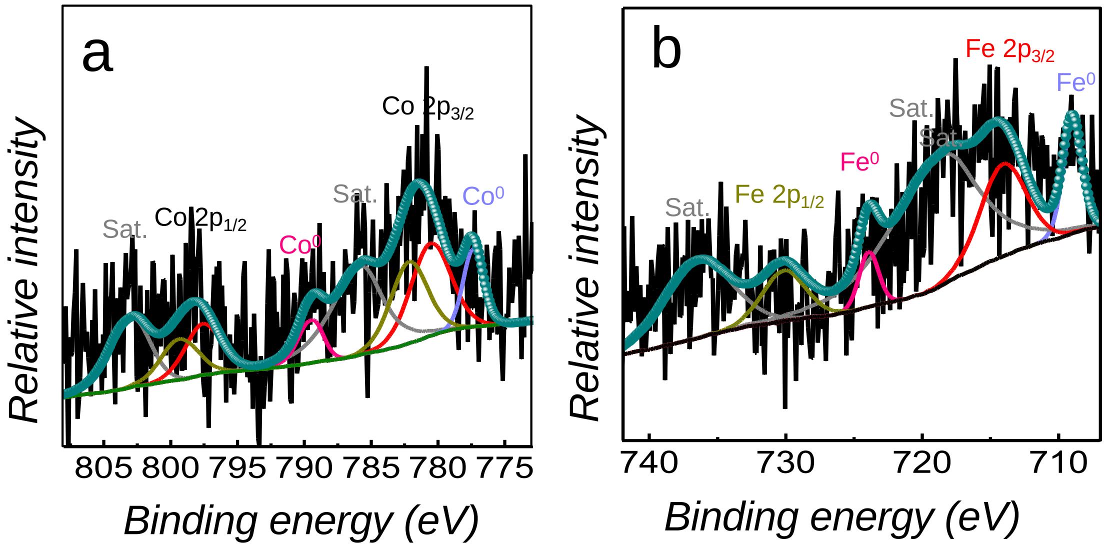

Supplementary Figure 2 | Characterization of PVP-C@LDO. a,b, Co 2p and Fe 2p XPS spectra of PVP-C@LDO.

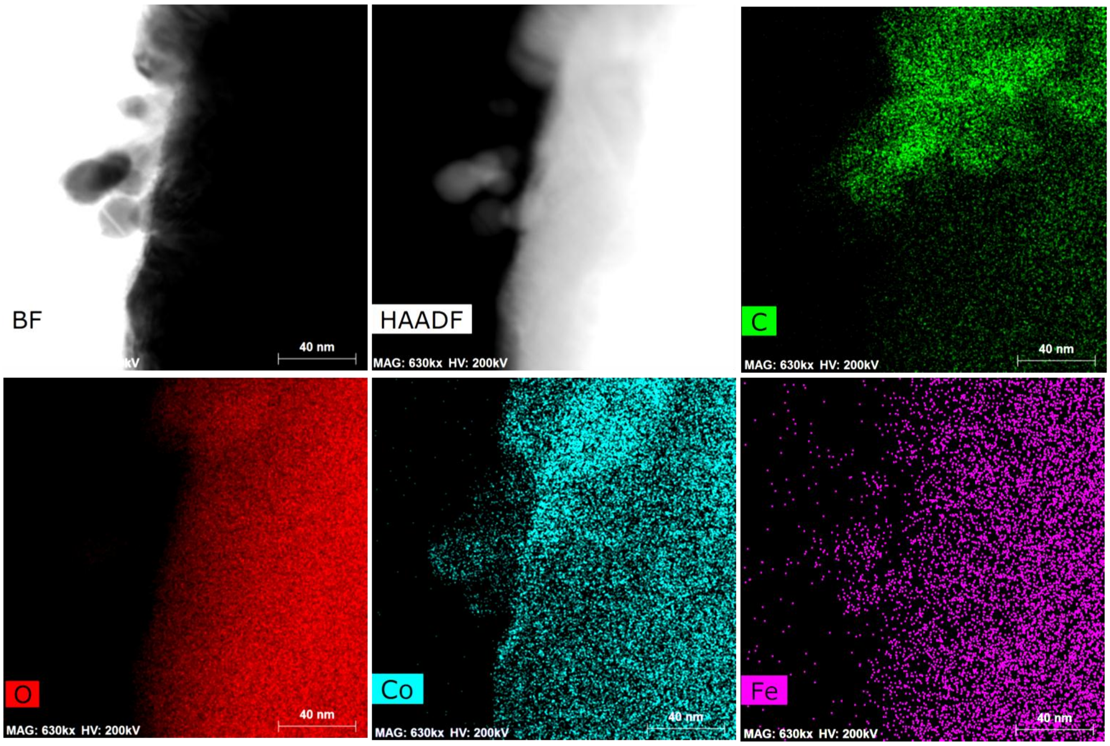

Supplementary Figure 3 | Elemental mapping images of PVP-C@LDO.

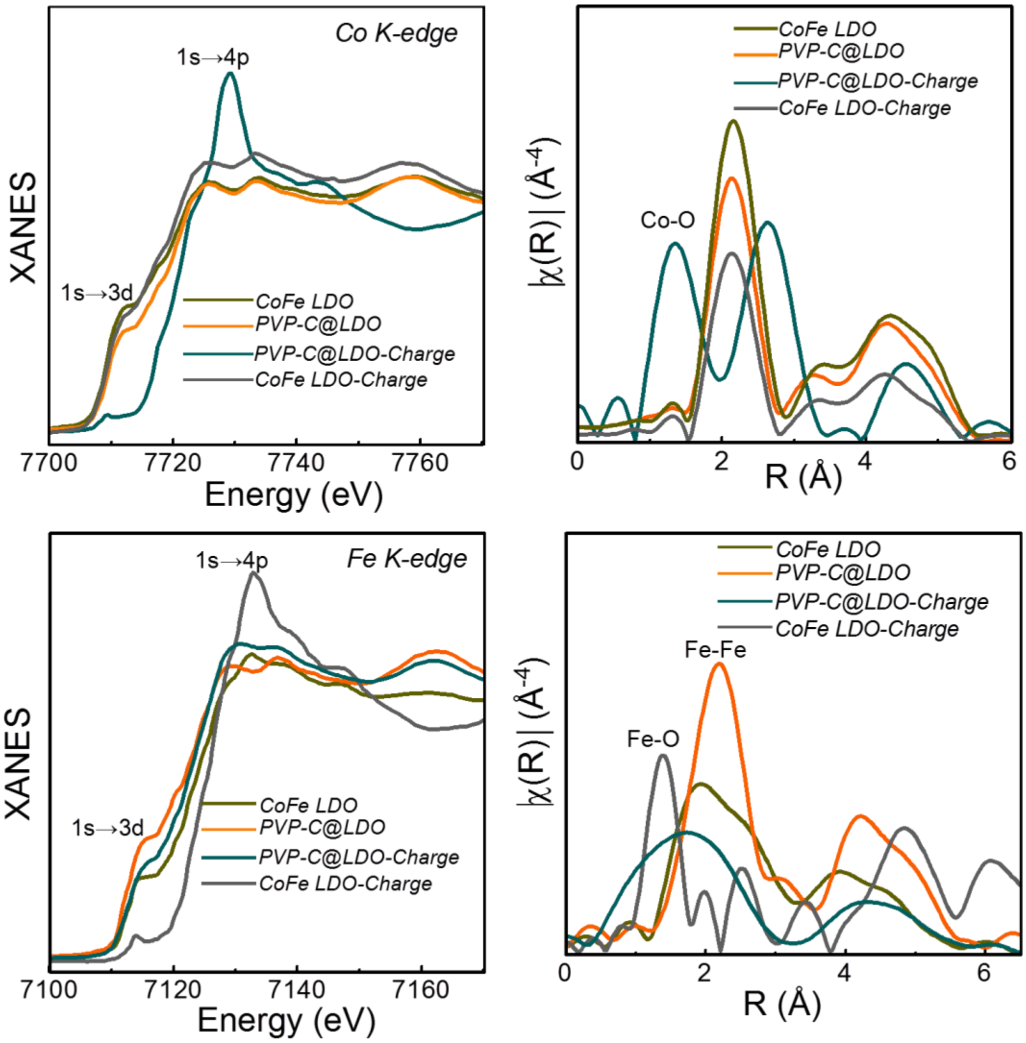

Supplementary Figure 4 | Co/Fe K-edge XAFS and EXAFS of CoFe LDO and PVP-C@LDO after first charge in conventional Li-O_2 batteries.

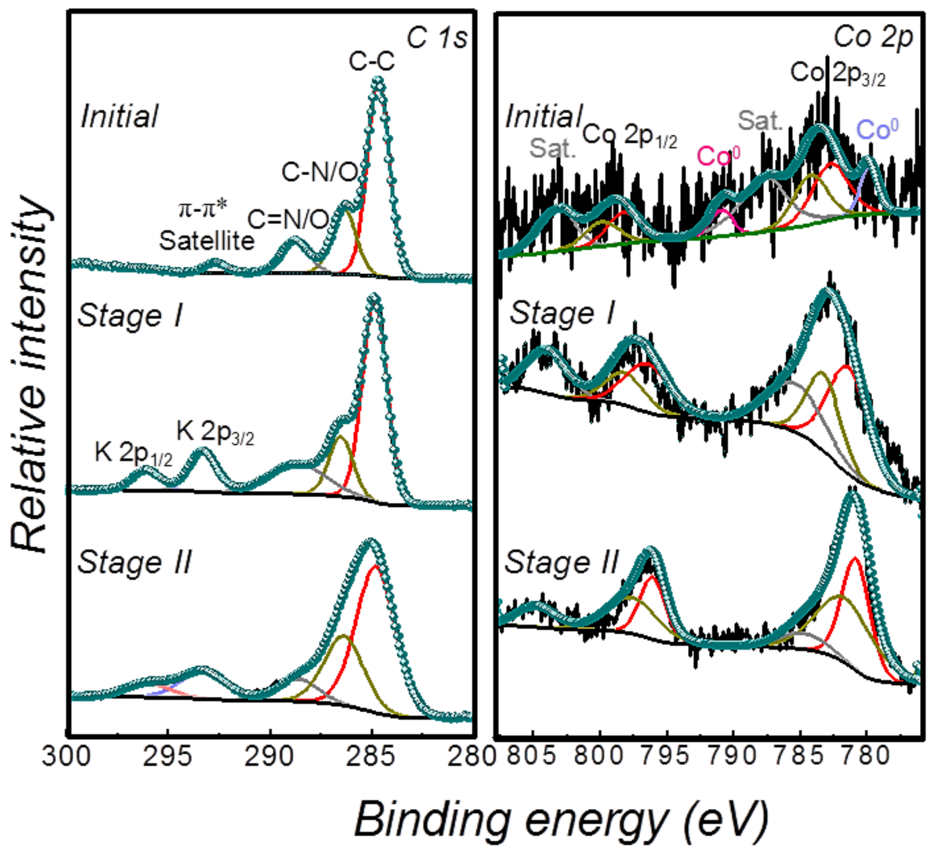

Supplementary Figure 5 | C 1s and Co 2p XPS spectra of initial PVP-C@LDO and discharged PVP-C@LDO at stage I and stage II.

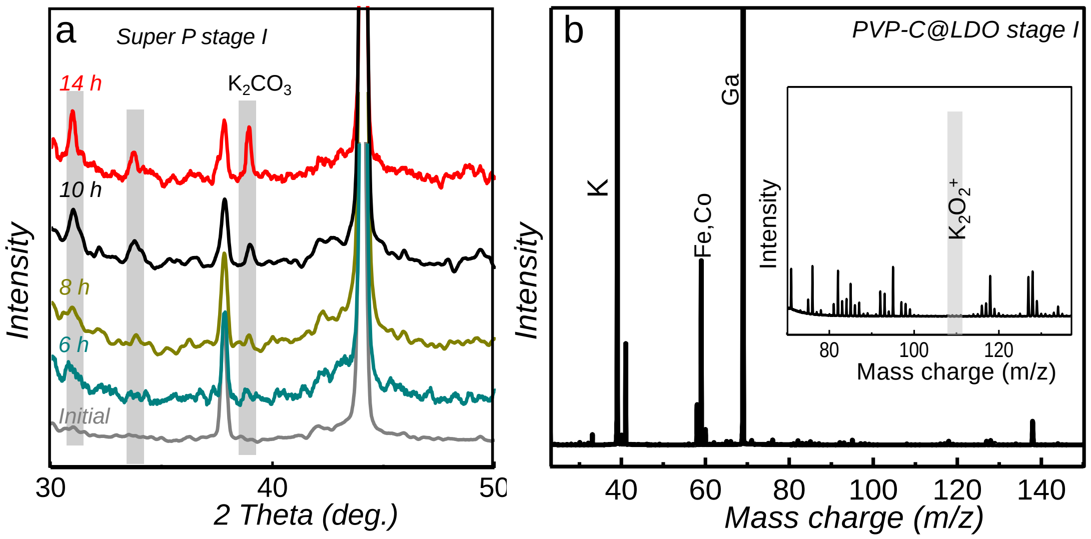

Supplementary Figure 6 | Discharge products at stage I. a, XRD patterns of discharged Super P cathodes with different time at stage I. **b**, Mass spectrum of discharge products on 1st discharged PVP-C@LDO at stage I.

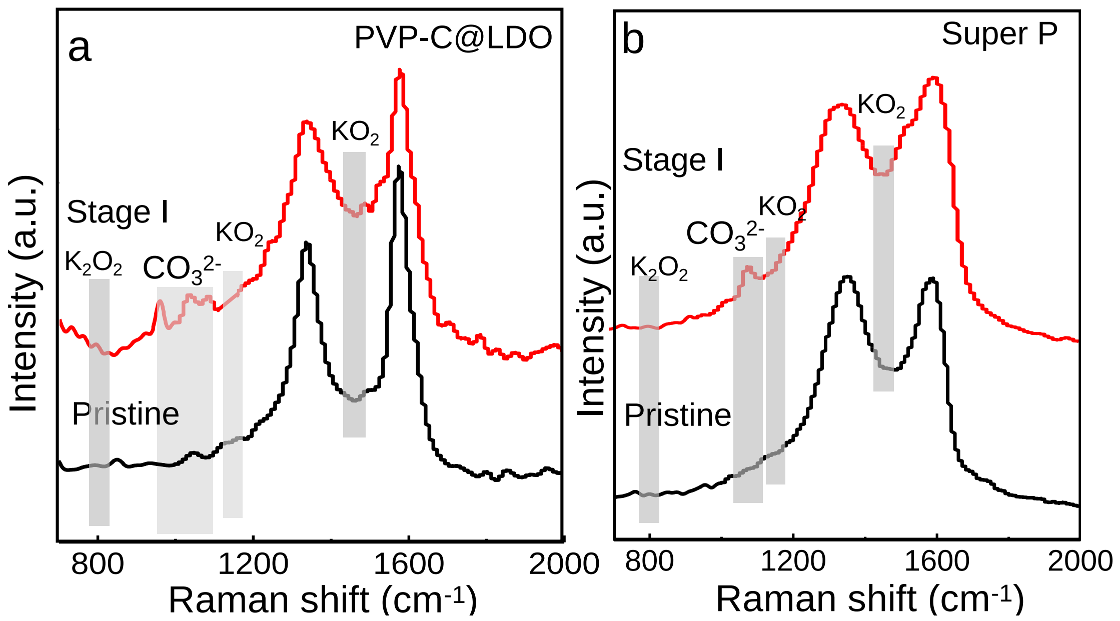

Supplementary Figure 7 | Discharge products at stage I. Raman spectra of **a**, PVP-C@LDO and **b**, Super P electrodes after discharge at stage I.

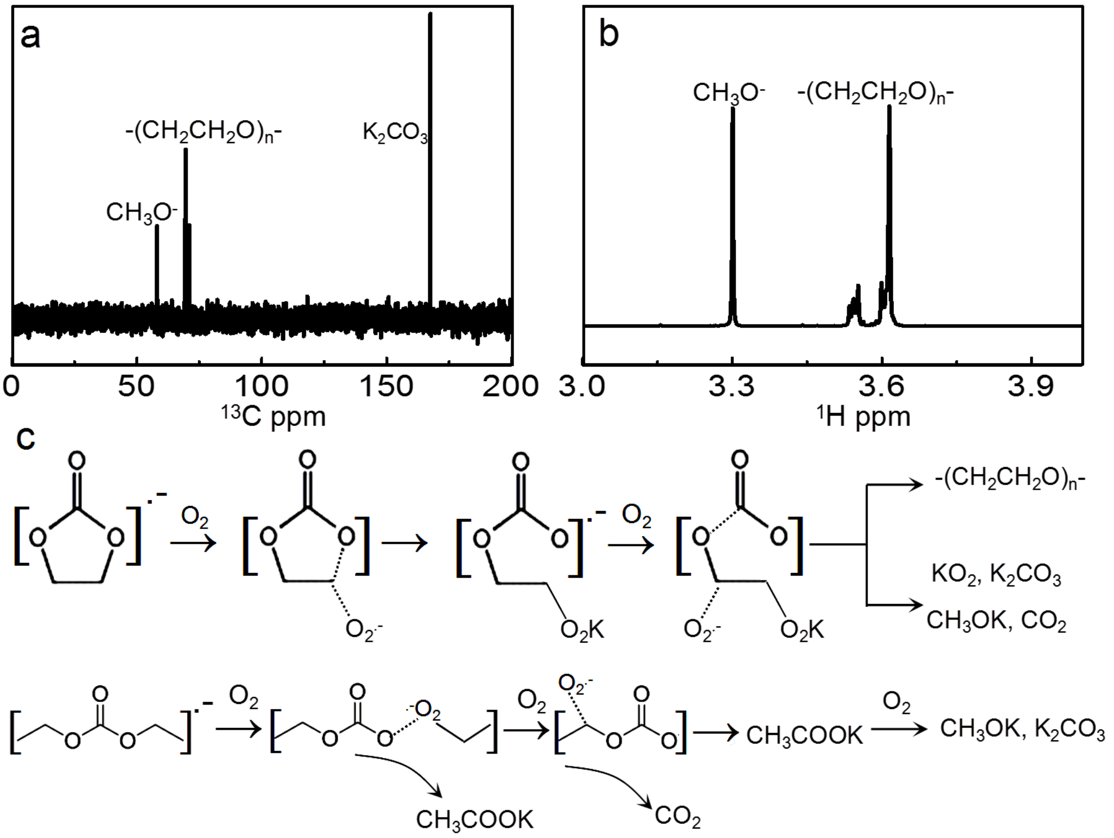

Supplementary Figure 8 | The formation mechanism of K_2CO_3 discharge products at stage I. a, ^{13}C-NMR and **b**, ^{1}H-NMR of the cathode surface layer at stage I in D_2O solution. **c**, Proposed mechanism of the formation of K_2CO_3 at stage I.

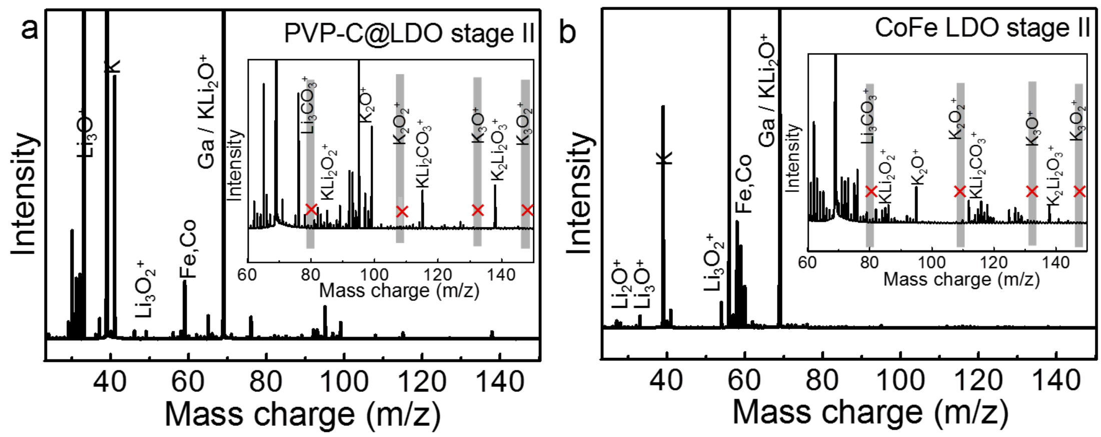

Supplementary Figure 9 | Mass spectra of discharged products at stage II. Mass spectra of discharged products on 1st discharged **a**, PVP-C@LDO and **b**, CoFe LDO cathodes surface at stage II.

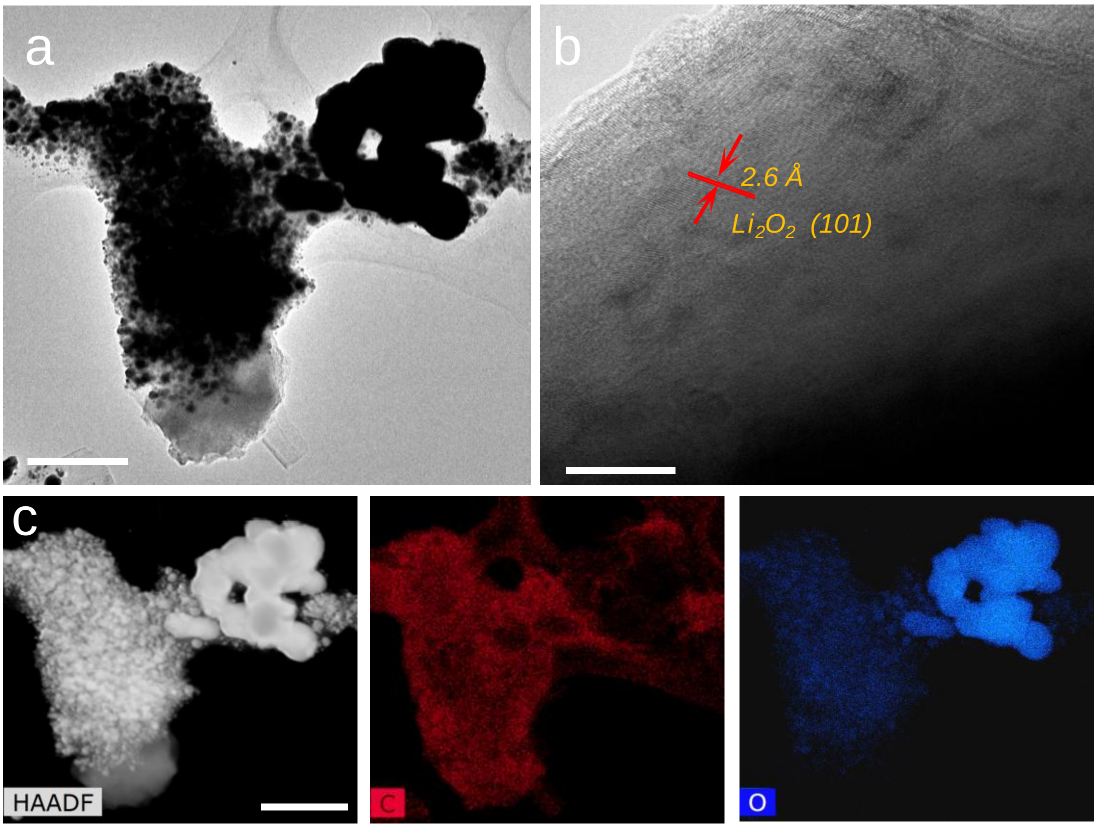

Supplementary Figure 10 | HRTEM and elemental mapping images of toroids in discharged PVP-C@LDO. Scale bars of **a-c** are 1 μm, 10 nm and 1 μm, respectively.

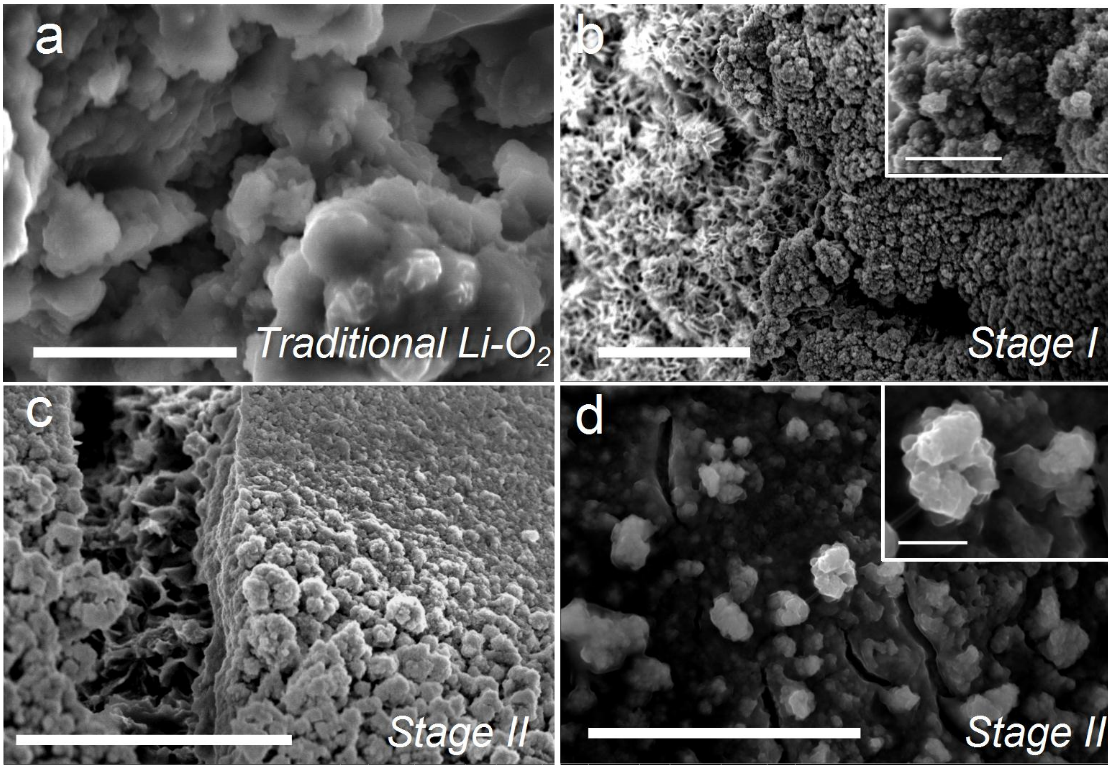

Supplementary Figure 11 | SEM images of discharged CoFe LDO in conventional Li-O_2 battery, and at stage I (K-O_2 battery), stage II (Li-O_2 battery). Scale bars of **a-d** are 5, 5, 5 and 4 μm, respectively. Scale bars of insets in **b** and **d** are 1 μm and 500 nm, respectively.

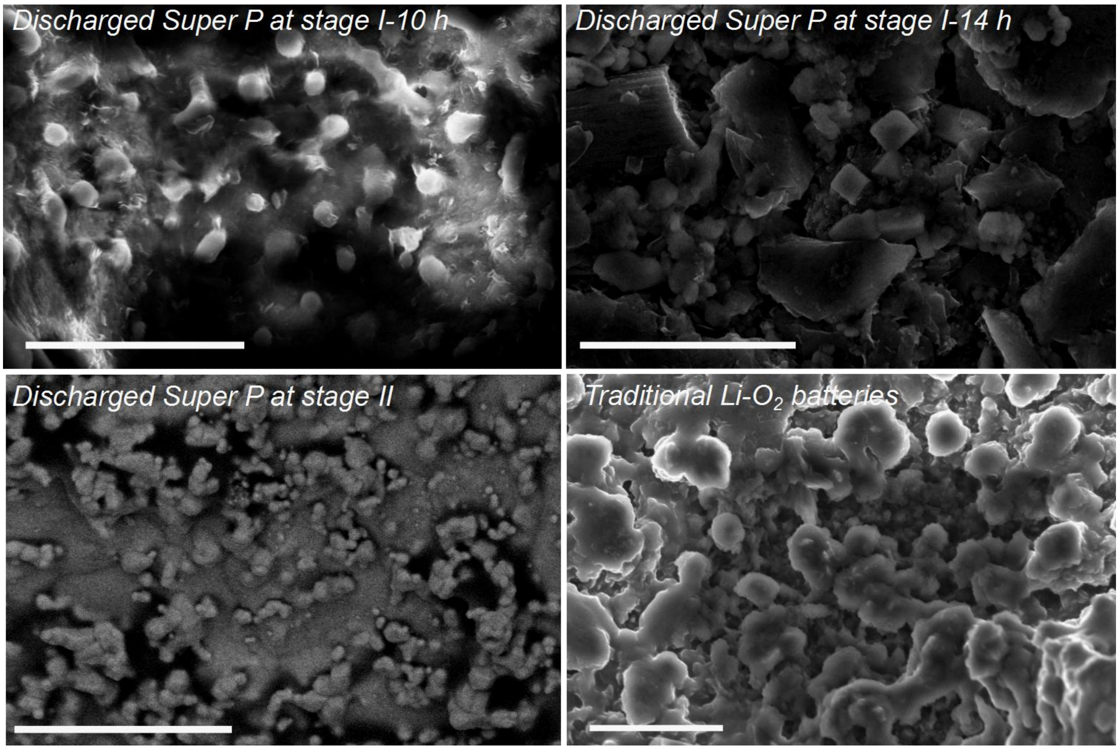

Supplementary Figure 12 | SEM images of Super P electrodes after discharge at stage I (10 h and 14 h), at stage II, and in conventional Li-O_2 batteries. Scale bars: 10 μm.

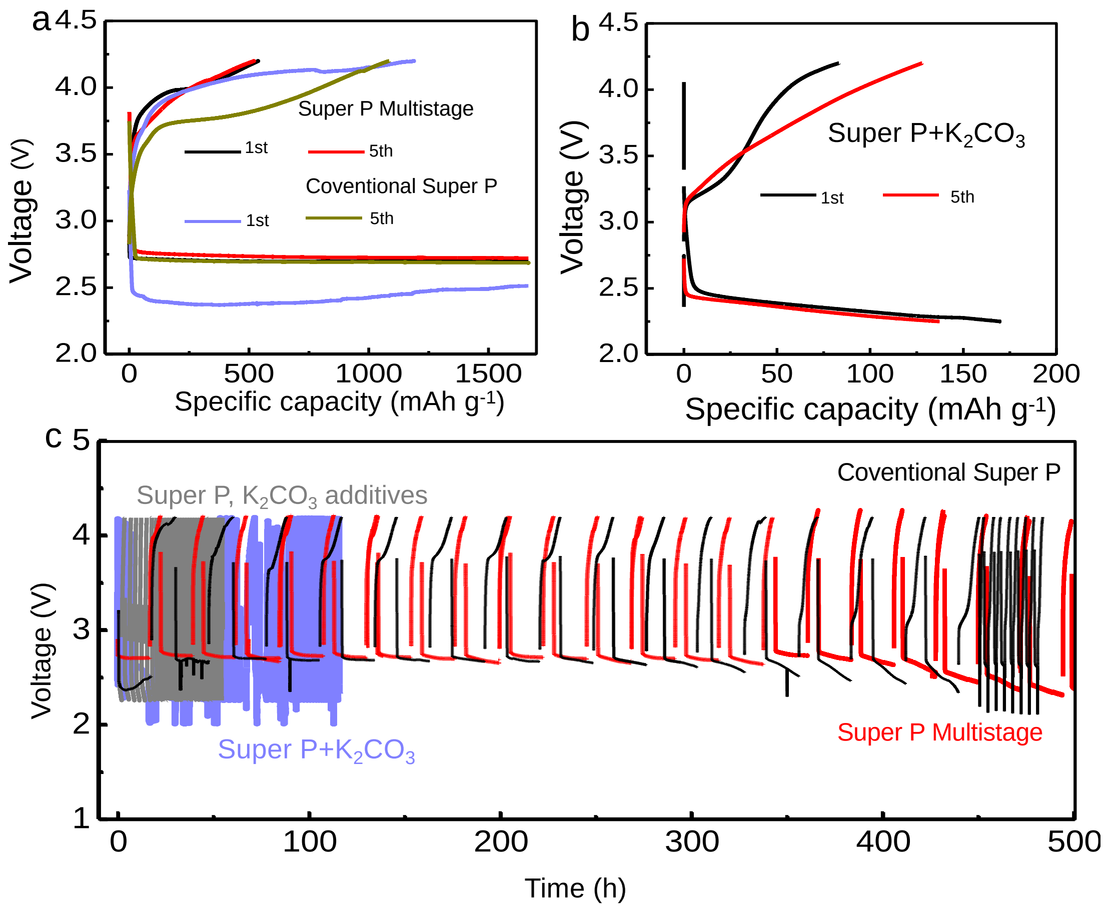

Supplementary Figure 13 | Electrochemical performance of Super P, Super P+K_2CO_3 (coated on the cathodes) and Super P with K_2CO_3 additive in electrolyte operated in O_2.

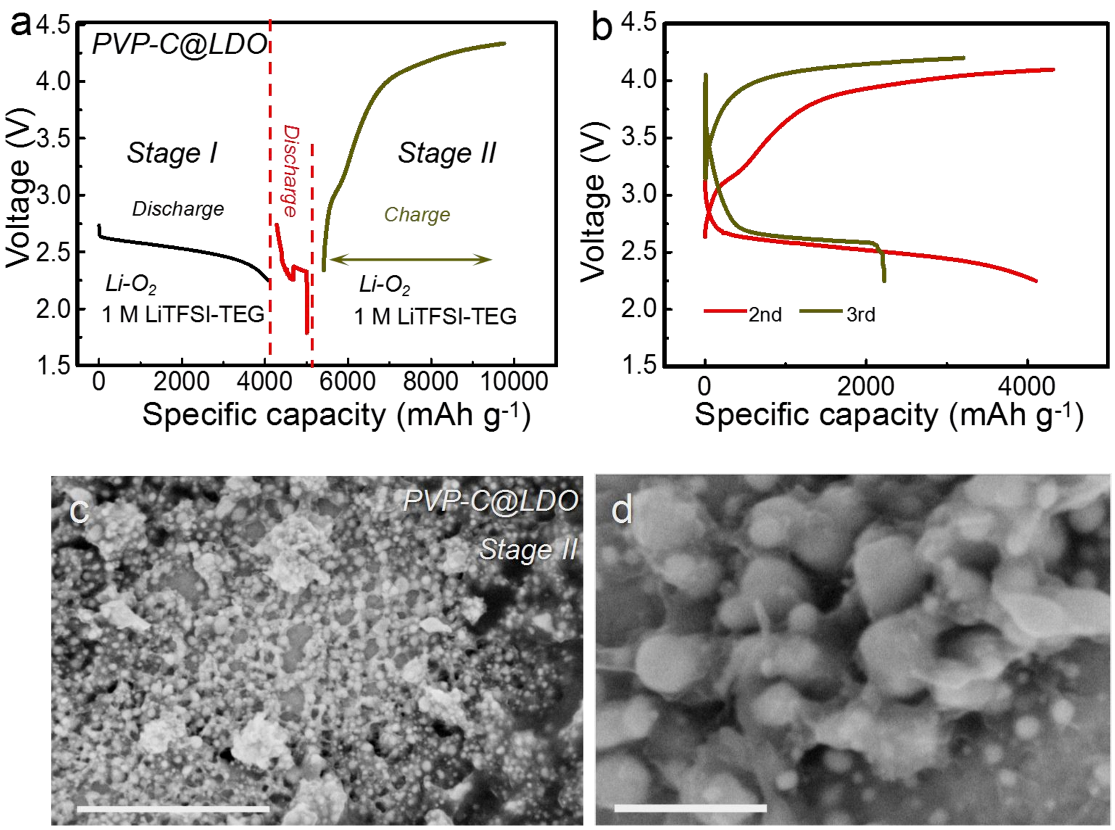

Supplementary Figure 14 | Disassembly of the first discharged PVP-C@LDO cathode in Li-O_2 battery and reassembly of the Li-O_2 battery with new electrolyte and separator. **a**, Multistaged pretreatment of PVP-C@LDO in Li-O_2 batteries. **b**, Cycling performance of PVP-C@LDO after multistaged discharge. Current densities are 100 mA g^{-1}. **c**,**d**, SEM images of discharged PVP-C@LDO at stage II in Li-O_2 batteries. Scale bars of **c**,**d** are 5 μm and 1 μm, respectively.

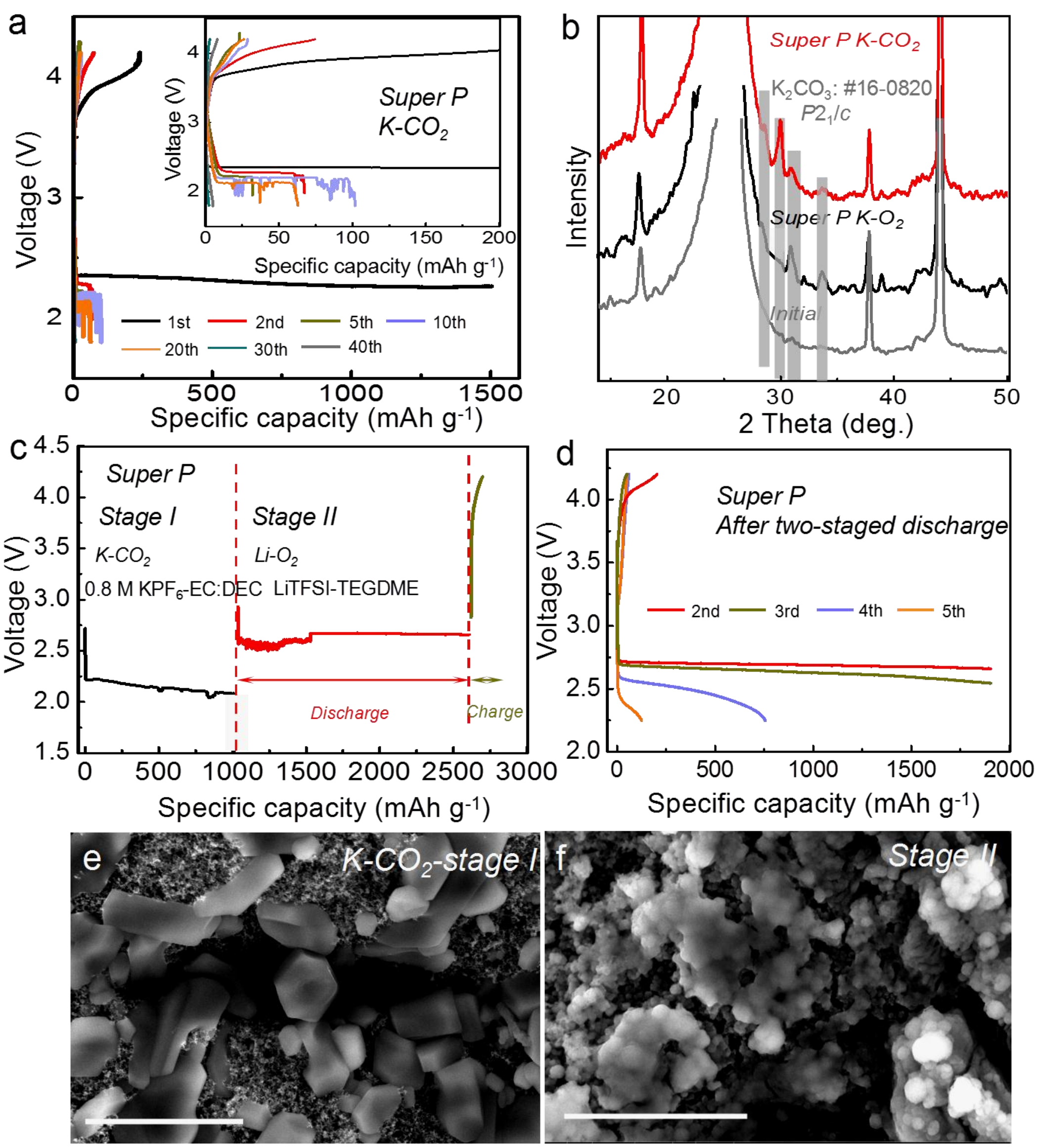

Supplementary Figure 15 | Two-staged discharge in K-CO_2 and Li-O_2 batteries. **a**, Cycle performance of Super P electrodes in K-CO_2 batteries. **b**, XRD patterns of discharged Super P at stage I in K-CO_2 battery and at stage I in K-O_2 battery. **c**, Two-staged discharge of Super P electrodes in stage I: K-CO_2 battery with EC: DEC as the electrolyte and stage II: Li-O_2 battery with TEGDME as electrolyte. **d**, Cycle performance of Super P electrodes after two-staged discharge. SEM images of discharged Super P **e**, at stage I (K-CO_2 battery); **f**, at stage II. Scale bars of **e**,**f** are 10 μm.

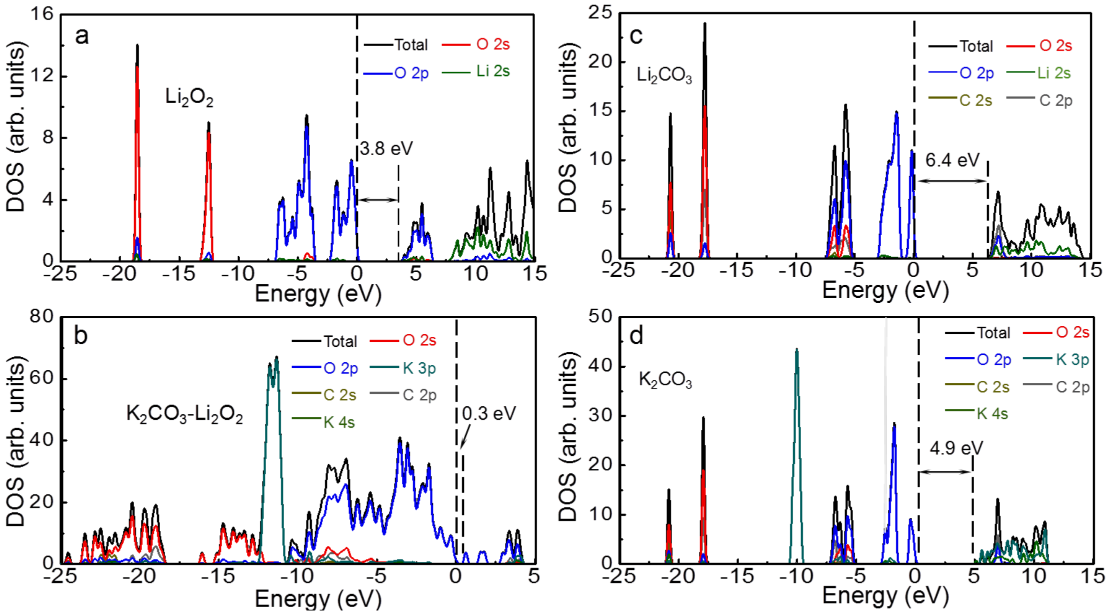

Supplementary Figure 16 | Band structure. Calculated densities of states (DOS), using GGA+U with U=6 eV, for **a**, Li_2O_2 ($P6_3/mmc$); **b**, K_2CO_3/Li_2O_2 heterostructure; **c**, Li_2CO_3 ($C2/c$); **d**, K_2CO_3 ($P6_3/mmc$).

Supplementary Table 1 **Electrochemical impedance spectroscopy (EIS) analyses of PVP-C@LDO before and after discharge at stage I and stage II**

**Samples**	R_u (Ohm)	R_{int} (Ohm)	R_{ct} (Ohm)	n	*W*-n
PVP-C@LDO	74.15	108.4	39.95	0.76	0.65
D-PVP-C@LDO Stage I	88.53	105.5	49.98	0.78	0.64
D-PVP-C@LDO Stage II	74.49	134.4	164.1	0.83	0.90
D-PVP-C@LDO-Li-O_2	31.95	329.9	192.1	0.66	0.53

Supplementary Table 2 **Structure details**

$P6_3/mmc$	Lattice parameters			Formation energy (eV)[1]	Density (g cm^{-3})[1]
	a	*b*	*c*		
K_2CO_3	5.48	5.48	8.03	-2.12	2.21
Li_2CO_3	4.65	4.65	5.38	-2.19	2.43
Li_2O_2	3.24	3.24	8.29	-1.65	2.26

Supplementary Note 1 - Valence states of Co, Fe after discharge in $Li-O_2$ battery

As shown in Supplementary Figure 4, after first charge in conventional $Li-O_2$ battery, XAFS spectra revealed the increase of valence states of Co and Fe in PVP-C@LDO, and EXAFS spectra revealed the presence of Co/Fe-O after 1st cycle. Co, Fe could be oxidized in strong oxidation environment in O_2 upon charge, which might result in the change of electrochemical performance of cathode catalysts.

Supplementary Note 2 - Side products at stage I

As shown in Supplementary Figure 8, CH_3OK (δ=3.33 ppm, singlet and δ=58.0 ppm), $-(CH_2CH_2O)_n-$ (δ=3.56, 3.62 ppm and δ=69.7, 67.6 ppm) can be identified in the ^{1}H-NMR and ^{13}C-NMR spectra of discharge products on Super P electrodes at stage I in D_2O. In Li-ion batteries, solvated Li^+ in EC electrolyte gaining electron will lead to the continuous reduction of EC to generate Li_2CO_3/CO_2[2,3]. The reductive decomposition of EC/DEC firstly generates semicarbonate by C-O cleavage. The semicarbonate might be further decomposed to release CO_2. The generated oligomer species were also reported in EC decomposition products in Li-ion batteries[4]. Neither CH_3OK (inorganic solid salt) nor $-(CH_2CH_2O)_n-$ (without conjugated structure) can construct heterostructure.

Supplementary Note 3 – The equivalent circuit and fitting of Nyquist plots

The equivalent circuit of PVP-C@LDO before and after discharge at stage I and stage II composed of three resistors and constant phase elements could be expressed as $R_u(R_{int}Q_{int})(C_d(R_{ct}Q_w))$. The R_u includes the ohmic resistance of electrolyte, and the electronic resistances of current collector and Li electrode. The R_{int} and Q_{int} correspond to the resistance and constant phase element of an interface layer, likely a cathode electrolyte interface (CEI) layer, respectively. The R_{ct} represents the charge-transfer resistance of the surface layer on the cathode in parallel with its double layer capacitance, C_d. The Q_w is the diffusion relative factor of Li^+. The deposition of discharge products on cathode surface significantly affected the R_{int} and R_{ct} values. The sum of R_{int} and R_{ct} of discharged PVP-C@LDO at stage II is far lower

than conventional discharge in Li-O_2 battery.

Supplementary References

1. Jain A. et al. Commentary: The materials project: A materials genome approach to accelerating materials innovation. *APL Mater.* **1**, 011002 (2013).
2. Xing, L. et al. Theoretical investigations on oxidative stability of solvents and oxidative decomposition mechanism of ethylene carbonate for lithium ion battery use. *J. Phys. Chem. B* **113**, 16596-16602 (2009).
3. Terborg, L. et al. Ion chromatographic determination of hydrolysis products of hexafluorophosphate salts in aqueous solution. *Anal. Chim. Acta.* **714**, 121-126 (2012).
4. Michan, A. L, Leskes, M. & Grey, C. P. Voltage dependent solid electrolyte interphase formation in silicon electrodes: monitoring the formation of organic decomposition products. *Chem. Mater.* **28**, 385-398 (2015).
